# Lobular Carcinoma of the Breast Metastatic to the Spleen and Accessory Spleen: Report of a Case

**DOI:** 10.1155/2016/5160180

**Published:** 2016-09-08

**Authors:** Gabriel M. Groisman

**Affiliations:** The Institute of Pathology, Hillel Yaffe Medical Center, Hadera, Israel

## Abstract

Despite the fact that accessory spleen (also known as supernumerary spleen, splenunculus, or splenule) can be found in 10–30% of patients undergoing autopsies, metastatic disease occurring in this organ has been barely reported. A case of lobular breast carcinoma metastatic to the spleen and accessory spleen found incidentally at therapeutic splenectomy for severe anemia and thrombocytopenia is described. On microscopic examination both organs revealed severe fibrocongestive changes and extramedullary hematopoiesis with no obvious carcinomatous involvement. Cytokeratin 7, estrogen receptors, and GATA3 immunohistochemistry disclosed the presence of numerous metastatic breast carcinoma cells infiltrating the splenic parenchyma. This case demonstrates that metastatic carcinoma can be encountered, although rarely, in accessory spleens and that cytokeratin stain should be performed in sections of spleens and/or accessory spleens excised from cancer patients in which the presence of malignant epithelial cells is not recognized on routine sections.

## 1. Introduction

Accessory spleen is a congenital anomaly in which there is failure of fusion between a portion of the developing splenic tissue and the main body of the spleen. Accessory spleens are present in approximately 10–30% of the population and are often an incidental finding [1–4]. Metastases from solid tumors to accessory spleens are extremely rare. To our knowledge only two such cases were reported previously [5, 6].

Infiltrating lobular carcinoma (ILC) represents about 5–15% of breast cancer cases [7]. It is well known that the pattern of metastatic spread of ILC is different from that of invasive ductal carcinoma, as the former may involve unusual locations such as the gastrointestinal tract, uterus, ovary, peritoneum, retroperitoneum, and meninges [7–10]. In addition, as ILC is composed of bland looking cells with a discohesive nature, its metastases can be missed in histological sections being discovered only when applying cytokeratin immunohistochemistry [11, 12].

In this report, we present the case of a patient with occult metastases of lobular carcinoma of the breast in the spleen and accessory spleen.

## 2. Case Presentation

**Figure 1:**
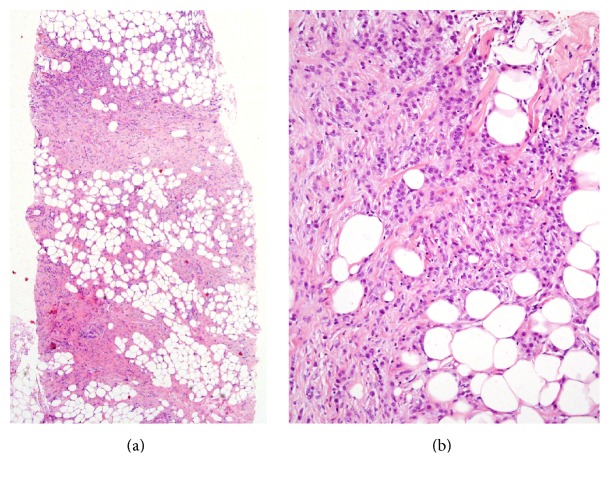
(a) Low power and (b) medium power microscopic views of the breast core biopsy showing invasive lobular carcinoma (hematoxylin and eosin stained section, magnifications ×40 and ×200 resp.).

The patient was a 49-year-old woman with a history of infiltrating lobular breast cancer. Two years before, a 4.0 cm, irregular, spiculated mass was detected on mammography. Core needle biopsies were performed and a diagnosis of infiltrating lobular carcinoma was made (Figures 1(a) and 1(b)). The tumor was found to be estrogen and progesterone receptors positive and HER2/neu negative and had a proliferation index of 15–25%. As subsequent clinical evaluation revealed the presence of cutaneous and bone marrow metastases (T2NXM1, stage IV) it was decided not to perform surgery and treatment with tamoxifen and goserelin was started. As a result of extensive bone marrow metastatic disease, she developed anemia, thrombocytopenia, and splenomegaly. Hypersplenism, caused by the splenomegaly, worsened the anemia and thrombocytopenia. As the hemoglobin and platelets counts decreased to 3.5 gr/dL and 8,000/mm^3^, respectively, and abdominal ultrasound demonstrated massive splenomegaly of size 27 cm., splenectomy was performed. At surgery, an accessory spleen was found in the splenocolic ligament and resected. One month following surgery, the hemoglobin and platelets counts reached 9.4 gr/dL and 213,000/mm^3^, respectively. Seven months following the procedure, the patient feels well and is being followed up by the division of hematology/oncology.

## 3. Pathological Examination

On gross examination the spleen measured 28 × 21 × 12 cm. and weighed 2,700 gr. whereas the splenunculus measured 3.0 cm. in diameter and weighed 6 gr. The capsules of both organs were smooth, tense, and intact and the parenchyma was dark red and firm with a vague nodular pattern. Representative sections were embedded in paraffin and stained with hematoxylin and eosin. Immunohistochemistry using the streptavidin-biotin peroxidase complex method was performed on a Ventana Benchmark automatic immunostainer (Tucson, AZ, USA) with the following antibodies: cytokeratin 7 (clone OV-TL12/30, ready to use [RTU]; Dako, Glostrup, Denmark), GATA3 (clone 634913, 1 : 50; R&D Systems, Minneapolis, MN, USA), estrogen receptor (clone SP1, RTU; Ventana, Tucson, AZ, USA), and E-cadherin (clone EP700Y, RTU; Cell Marque, Rocklin, CA, USA).

**Figure 2:**
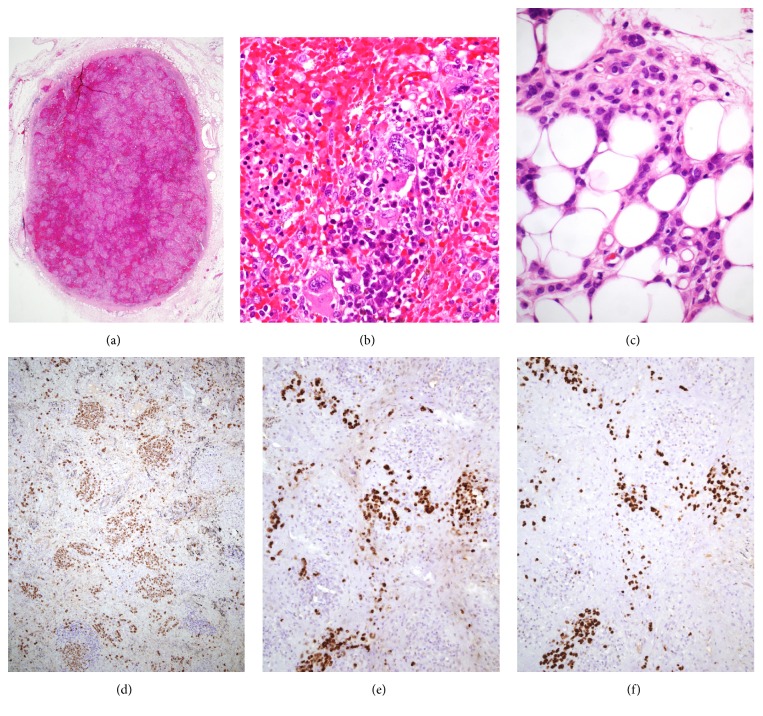
(a) Low power microscopic view of the accessory spleen showing marked congestion and a nodular pattern (hematoxylin and eosin stained section, magnification ×20). (b) High power view shows extramedullary hematopoiesis including atypical megakaryocytes (hematoxylin and eosin stained section, magnification ×400). (c) Small cells with occasional cytoplasmic vacuoles resembling lobular carcinoma cells infiltrate the fatty tissue (hematoxylin and eosin stained section, magnification ×400). (d) Cytokeratin stain highlights numerous cells that represent metastatic carcinoma (magnification ×100). (e) Estrogen receptors and (f) GATA3 strongly stain the nuclei of the cancer cells confirming the mammary origin of the cancer ((e) and (f) magnification ×200).

Histologic sections of both the spleen and the splenunculus revealed inconspicuous white pulp and marked expansion of the red pulp due to marked congestion and extensive extramedullary hematopoiesis (EMH) distributed in a nodular pattern (Figure 2(a)). Myeloid and erythroid elements infiltrated cords and sinuses and megakaryocytes occasionally formed clusters that included forms with bizarre morphologic features (Figure 2(b)). Foci of EMH were separated by bands of reticulin and fibrous connective tissue. In addition, there was severe blood congestion, accumulation of hemosiderin-containing macrophages, and formation of fibroelastotic nodules with hemosiderin deposition (“Gamna-Gandy bodies”).

The presence of metastatic carcinoma within the splenic parenchyma could not be definitely established from hematoxylin and eosin stained sections. However, small cells with occasional cytoplasmic vacuoles resembling lobular carcinoma cells were detected within the adjacent fatty tissue (Figure 2(c)). Immunohistochemically, these cells, as well as numerous additional cells widely distributed within the spleen and accessory spleen parenchymata as single units or small, loose aggregates reacted strongly with cytokeratin 7, estrogen receptor, and GATA3 (Figures 2(d), 2(e), and 2(f)). In contrast, E-cadherin stain was negative. These results supported the diagnosis of lobular breast carcinoma metastatic to the spleen and accessory spleen.

## 4. Discussion

The occurrence of breast carcinoma metastasizing to an accessory spleen has not been reported yet. In this case, clinically unsuspected metastatic lobular breast carcinoma cells diffusely infiltrated the spleen and the accessory spleen. Microscopically, metastatic carcinoma was not clearly identified with hematoxylin and eosin stained sections although suspicious cells were present within the adjacent fatty tissue. Immunohistochemical stains, however, disclosed the presence of numerous metastatic lobular carcinoma cells within the spleen and accessory spleen parenchymata and confirmed the malignant nature of the suspicious cells observed in the adipose tissue adjacent to the spleens. These findings demonstrated that the severe splenomegaly resulted not only from severe congestion and EMH but also from metastatic carcinoma.

Accessory spleens, also known as supernumerary spleens, splenunculi, or splenules, are congenital foci of healthy splenic tissue that are separate from the main body of the spleen [1]. They arise from the failure of fusion of the splenic anlage, located in the dorsal mesogastrium, during the fifth week of fetal life [2]. Accessory spleens are relatively common and are seen in 10–30% of patients at autopsy [1–3].

Metastases from solid tumors to accessory spleens are extremely rare. To our knowledge only two such cases were reported previously. Mihmanli et al. [5] described a case of ovarian carcinoma metastatic to a mass located in the transverse mesocolon which resulted to be an accessory spleen containing metastatic ovarian carcinoma. The second case, reported by Porwal et al. [6], was a retroperitoneal, suprarenal accessory spleen infiltrated by metastatic transitional cell carcinoma of the kidney.

Metastatic carcinoma of the spleen is an uncommon clinical problem [13]. Most splenic metastases develop in patients with multivisceral metastatic disease being skin melanoma and breast, lung, ovarian, colorectal, and gastric carcinomas the most common primary sources [14, 15]. Rarely, breast carcinoma metastatic to the spleen may present as solitary splenic metastatic masses [16–19] or associated with idiopathic (immune) thrombocytopenic purpura [20]. The relative paucity of splenic epithelial metastases may be a result of proapoptotic signals in the spleen, leading to decreased epithelial survival [21].

Metastatic lobular carcinoma of the breast, unlike the ductal type, can be difficult to recognize in routine histologic sections [11]. Bland nuclear features, a discohesive infiltration pattern, and the frequent lack of a desmoplastic stromal reaction contribute to this difficulty. Moreover, the occurrence of occult lymph node metastasis from breast cancer has been estimated to be about 20%, with a rate as high as 39% being reported in lobular carcinoma [12]. Likewise, Lyda et al. [22] documented that keratin immunohistochemistry detected clinically significant bone marrow metastases of lobular breast carcinoma in which the initial histologic examination was negative.

In the present case both the spleen and accessory spleen displayed severe congestion, extramedullary hematopoiesis (EMH), and marked nodular fibrosis. Carcinoma cells were not evident on hematoxylin and eosin stained sections and were only disclosed with cytokeratin stain. To confirm the mammary origin of the cancer cells we employed estrogen receptors and GATA3 immunostains. The latter is a novel transcription factor belonging to the GATA family, proved to be a useful immunohistochemical marker for several malignancies, mainly breast and urothelial carcinomas [23, 24]. Miettinen et al. [23] found that 92% and 96% of primary and metastatic mammary ductal carcinomas, respectively, and 100% of mammary lobular carcinoma were diffusely positive for this marker. However, as other tumors such as chromophobe renal cell carcinoma, basal cell carcinoma, and mesothelioma have been reported to be reactive to GATA3 [24], it is advisable to use more than one marker to confirm the mammary origin of a metastatic carcinoma.

In summary, to our knowledge this is the first report of breast carcinoma metastasizing to an accessory spleen. It underscores the importance of getting a comprehensive patient history and conducting immunohistochemical stains in relevant cases to avoid missing a diagnosis of metastatic carcinoma.
